# Fibrolipoma Causing Carpal Tunnel Syndrome

**DOI:** 10.5334/jbsr.2601

**Published:** 2021-09-27

**Authors:** Anthony Timmerman, Filip Vanhoenacker, Lucas Walschot

**Affiliations:** 1 AZ Sint-Maarten, Mechelen and University (Hospital) Leuven, BE; 2 AZ Sint-Maarten, Mechelen and University (Hospital) Antwerp/Ghent, BE; 3 AZ Sint-Maarten, Mechelen, BE

**Keywords:** fibrolipoma, carpal tunnel syndrome, median nerve, magnetic resonance imaging, ultrasound

## Abstract

**Teaching point:** A space-occupying lesion should be considered in the differential diagnosis of progressive unilateral carpal tunnel syndrome.

## Case Presentation

A 56-year-old women presented with long-lasting, radial-sided paraesthesia in her left hand, having suffered from a conservatively treated ipsilateral distal radius fracture six years prior. A mass was palpable in the palm of the hand, which moved proximally under the transverse carpal ligament upon flexion of the fingers accompanied by a painless “snap” (trigger phenomenon). Carpal tunnel syndrome (CTS) provocative tests were positive, and electromyography (EMG) confirmed CTS.

**Figure 1:**
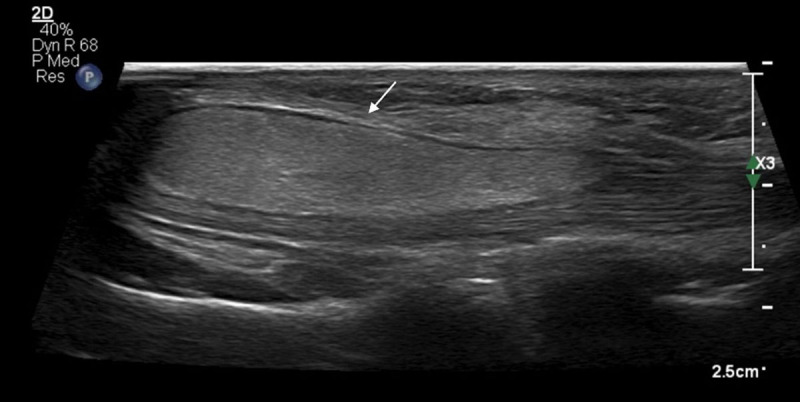


**Figure 2:**
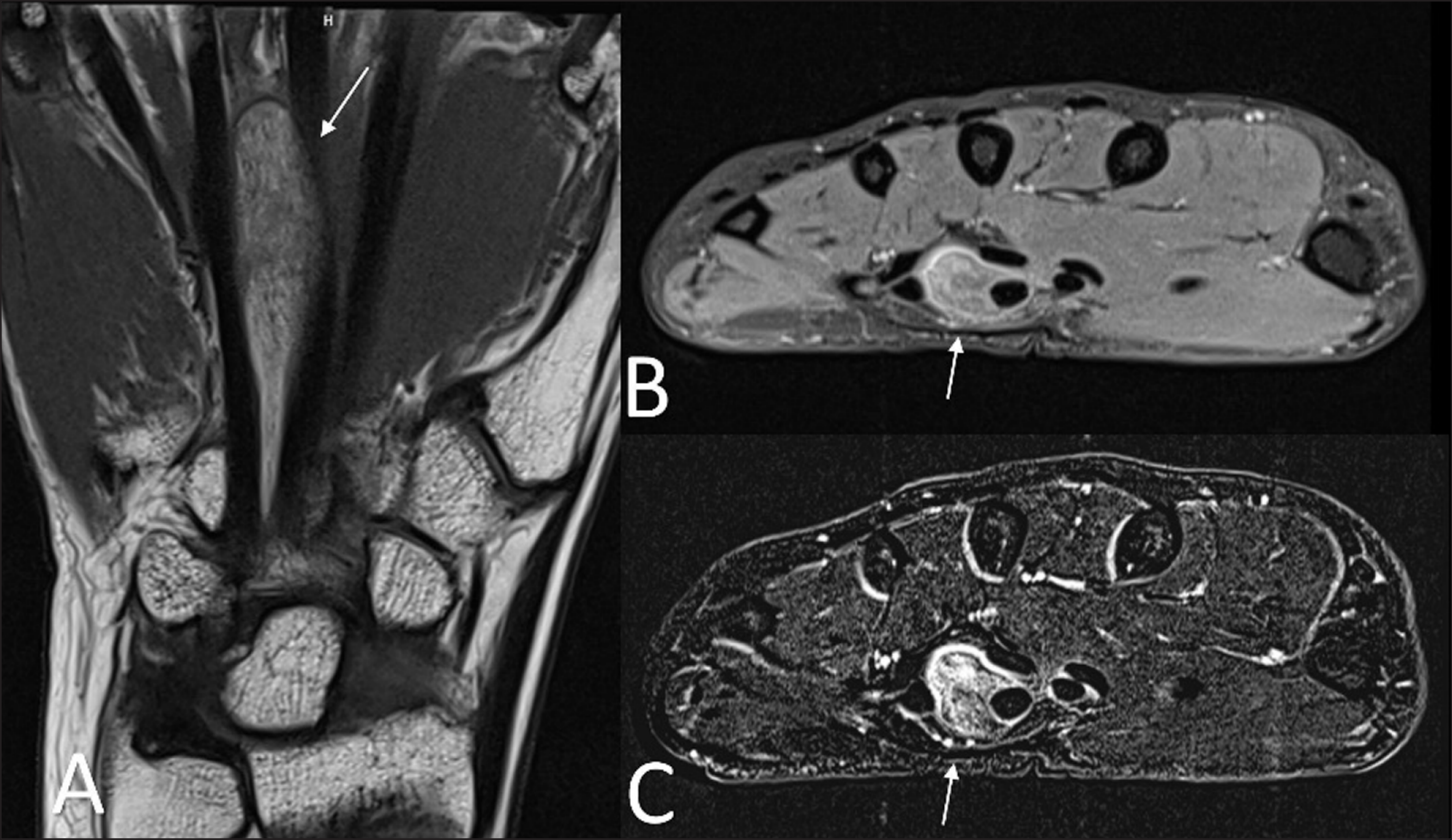


**Figure 3:**
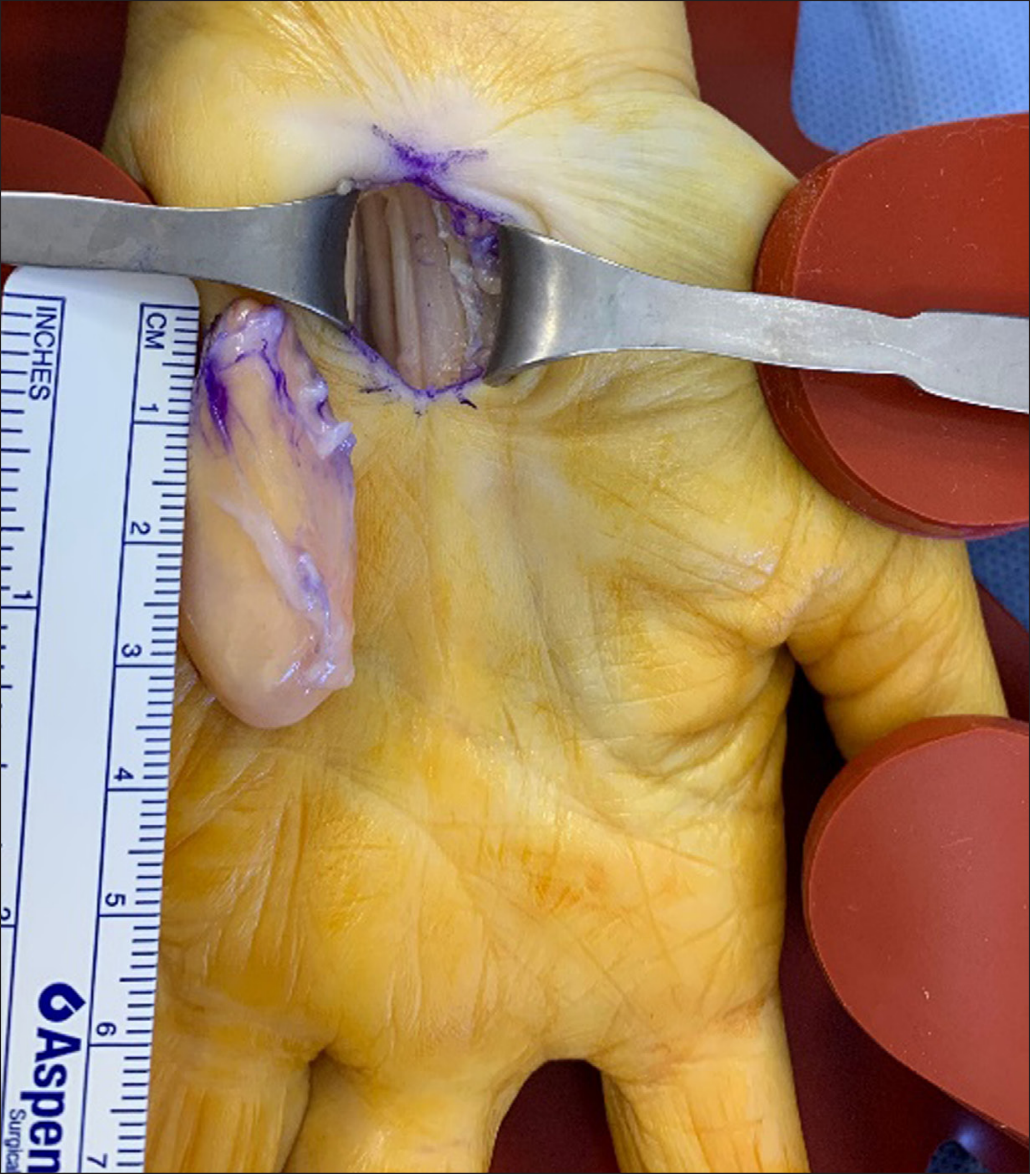


Conventional radiography was unremarkable. Ultrasound (US) showed a well-delineated fusiform lesion at the ulnar side of the carpal tunnel, extending between the third and fourth metacarpal. The lesion was homogeneously hyperechoic with absence of increased intralesional doppler signal (***Figure 1***, arrow). On magnetic resonance imaging (MRI), the signal intensity was similar to that of subcutaneous fat, except for thin intralesional strands of non-lipomatous tissue, showing enhancement (***Figure 2A***; coronal T1-weighted, ***2B***; axial fat-suppressed T1-WI, ***2C***; axial subtraction image of the fat-suppressed T1-WI before and after administration of gadolinium contrast, arrows). The preferential diagnosis of fibrolipoma was made, although an atypical lipoma could not be excluded. Resection followed by histopathological examination showed multiple adipocytes with absence of atypical cells and fibrous bands were seen in between, confirming the diagnosis of a fibrolipoma (***Figure 3***; peroperative specimen).

## Discussion

A lipoma is a benign tumour, originating from adipose tissue. Although it is the most common soft tissue tumour in the body, it is rarely seen in the hand [1]. They are one of the uncommon benign tumours that can arise from the synovial membrane of a tendon sheath. Differential diagnosis contains fibrolipomatous hamartoma (neural fibrolipoma), often on the course of the nerve but also malignant adipocytic tumours such as atypical lipomatous tumour (ALT), which is locally aggressive, or liposarcoma. Trauma has been related with lipomas, although this association remains highly debatable and the etiologic mechanism unclear. Post-traumatic lipoma formation could be due to preadipocyte differentiation mediated by cytokine release or prolapse of adipose tissue through fascia by direct impact.

CTS usually presents as a bilateral disorder. In case of long-standing unilateral symptoms, secondary causes due to a space-occupying lesion should be suspected. Further work-up with EMG, US, and MRI is needed for detection and characterization of the lesion [1]. Although the diagnosis of a usual lipoma is straightforward if the lesion is homogeneously isointense to fat, ALT cannot be excluded if the lesion contains non lipomatous components. Complete lesion excision and histological examination remains necessary to confirm the diagnosis. Conventional open approach leads to good results and is recommended above endoscopic methods. Local recurrence is rare, and no strict follow-up regimen is required [1].
